# Temperature-Dependent Cytokine Neutralization Induced by Magnetoelectric Nanoparticles: An In Silico Study

**DOI:** 10.3390/ijms252413591

**Published:** 2024-12-19

**Authors:** Alessandra Marrella, Paolo Giannoni, Martina Lenzuni, Giulia Suarato, Serena Fiocchi, Emma Chiaramello, Paolo Ravazzani

**Affiliations:** 1 Institute of Electronics, Computer and Telecommunication Engineering (IEIIT), National Research Council of Italy (CNR), 20133 Milan, Italy; martina.lenzuni@cnr.it (M.L.); giulia.suarato@cnr.it (G.S.); serena.fiocchi@cnr.it (S.F.); emma.chiaramello@cnr.it (E.C.); paolo.ravazzani@cnr.it (P.R.); 2 Department of Experimental Medicine, Biology Section, University of Genoa, 16132 Genoa, Italy; paolo.giannoni@unige.it

**Keywords:** magnetoelectric nanoparticles, multiphysics modelling, inflammatory cytokines, biological neutralization

## Abstract

Inflammatory cytokines cooperate to maintain normal immune homeostasis, performing both a protective and a pro-inflammatory action in different body districts. However, their excessive persistence or deregulated expression may degenerate into tissue chronic inflammatory status. Advanced therapies should be designed to deploy selective cytokine neutralizers in the affected tissues. Magnetoelectric nanoparticles (MENPs) possess unexploited potentialities, conjugating their ferromagnetic nature, which enables confinement in a specific tissue by directed positioning when subjected to low-intensity magnetic fields, with the capability to generate high electric fields with elevated spatial resolution when subjected to higher magnetic fields. This work proposes to exploit the extremely localized heat generated by Joule’s effect around MENPs under an external magnetic field to denature a harmful cytokine in a hypothetical tissue site. An interdisciplinary and multiphysics in silico study was conducted to provide comprehensive modeling of the temperature distribution generated by MENPs decorated with a membrane-derived microvesicle (MV) coating designed to allocate a specific antibody to bind a target cytokine. A damage model was also implemented to provide an estimation of the influence of several design parameters on the cytokine denaturation efficacy, with the final goal of guiding the future development of effective MENPs-based therapeutic applications and strategies.

## 1. Introduction

Inflammation is characterized by an excessive production of inflammatory cytokines in response to various agents, such as pathogens, autoimmune diseases, different types of cancer, and even chemicals used in long-term therapies. In normal conditions, cells of the immune system secrete inflammatory cytokines, such as interleukin-1 (IL-1), interleukin-6 (IL-6), interleukin-8 (IL-8), tumor necrosis factor-α (TNF-α), and others [1], which trigger healing mechanisms and cooperate to restore and maintain the immune homeostasis. However, if an overproduction of these cytokines occurs, the so-called pro-inflammatory “cytokine cascade” arises, leading to further inflammation mechanisms. Moreover, even different cancer immunotherapeutic approaches can upregulate the production of inflammatory cytokines, initiating a process called “immune-related adverse events (irAEs)”.

Although cytokine inhibitors and, more in general, anti-inflammatory agents have already reached the clinical scenario, their resulting efficacy is limited due to the toxicity of the high systemic dosages and their rapid clearance out of the body [2]. Moreover, systemic inhibition of inflammatory cytokines may lead to the onset and recurrence of several adventitious pathologies; for example, different types of infections have been reported in patients treated with compounds that inhibit cytokines (i.e., anti-TNF agents), including severe pneumonia [3,4], meningitis [5], sepsis [6], histoplasmosis [7], and aspergillosis [8].

The severely harmful role of an excess of inflammatory cytokines has prompted researchers involved in the bio-nanotechnology field to develop new therapeutic agents in the form of nano-vectors, such as nanoparticles conjugated with cytokine-neutralizing antibodies, with the aim to reach tissue areas poorly accessible by free antibodies, widening the range of therapeutic efficacy, and to improve their (i) retention after administration, (ii) stability, and (iii) specific targeting. One of the currently performed strategies relies upon the decoration of the nanoparticle surface with cell-derived membranes to facilitate cytokine binding as well as tissue compatibility [9,10,11]. Depending on the cell source, coatings may present several receptors, allowing cytokine neutralization without aiming at a specific target.

Recently, magnetic inorganic nanoparticles have emerged as nano-tools to capture different molecules involved in the regulation and functioning of the immune systems [12]. They are particularly suitable for minimally invasive magnetic-controlled guidance within the human circulatory system. In fact, they can be adapted to drag a molecule—either an antibody or a drug—to a specific tissue site; thus reducing the possible systemic side effects on healthy tissues and improving localized clinical efficiency.

Magnetoelectric nanoparticles (MENPs) differ from well-known magnetic nanoparticles because of their significant magnetoelectric (ME) properties, which enable the generation of local electric fields when external magnetic ones are remotely applied and vice versa [13]. The most efficient configuration in terms of magnetoelectric coupling is a core-shell structure, where a magnetostrictive core composed of Cobalt Ferrite (CoFe) and a piezoelectric shell of Barium Titanate (BaTi) are coupled. According to previous results [14,15], when an external DC bias is activated, MENPs generate a local electric field that can reach values above 10^5^ V/m and that decays very quickly with the distance from the MENP surface. Such high electric field intensity generates a power density that induces local heating, which is deployed in the surrounding milieu with the same rapid spatial decay of the electric field. In the present study, the MENPs potential to produce high temperatures in an extremely localized area is exploited with the final aim of denaturing target cytokines without damaging bystander cells and tissues. The driving idea is that MENPs can be decorated with a biological coating, for example, composed of cell-derived microvesicle membranes (MV) onto which a specific antibody(ies) could be linked to catch specific molecules. Once the antibody-cytokine binding occurs, then MENPs can be activated to thermally neutralize the target cytokine without any damage occurring to the surrounding healthy tissues, thanks to their highly spatially confined magnetoelectric effect. MV-coating and encapsulation procedures, in fact, provide a biological camouflage, with respect to bare particle surfaces, that can help to avoid interactions with the immune system reactants in the bloodstream, which may lead to the particle vector degradation by the endo/lysosomal compartment [16,17].

The current study presents an in silico interdisciplinary modeling of the MENPs electrothermal features when stimulated by external DC fields to provide a priori knowledge of the in vivo denaturation capability of such decorated nano-systems. Indeed, presented data allow us to predict the heat damage occurring on target cytokines, bonded on the MV-coated surface of MENPs, by using the Arrhenius thermal damage model, currently adopted for the thermal dosage assessment in different clinical therapies [18]. As a whole, this modeling approach and the relative array of derived parameters may represent a valuable benchmark to foster MENPs future therapeutic applications.

## 2. Results

**Figure 1:**
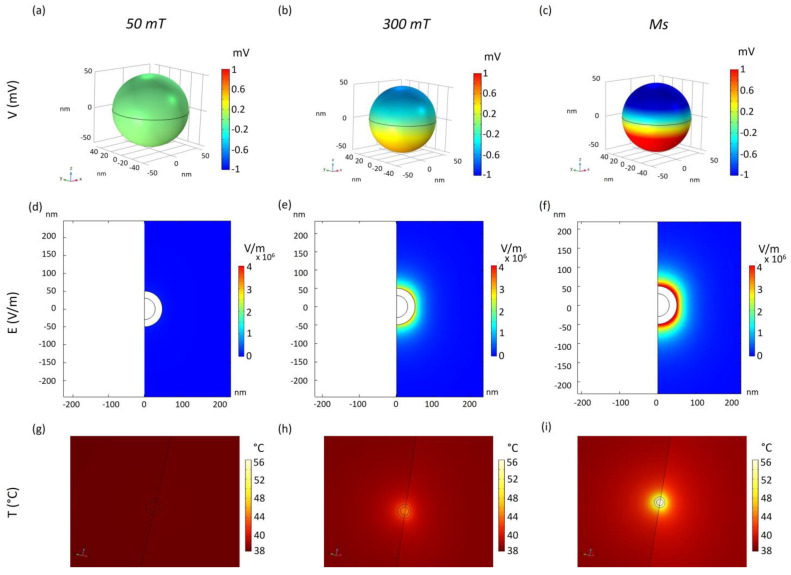
ME-guided temperature increase. Three-dimensional distribution of electric potential V(mV) over the 3D CoFe/BaTi core-shell MENPs (**a**–**c**), the electric field distribution E(V/m) within the surrounding extracellular fluid (**d**–**f**), and the consequent localized temperature increase (**g**–**i**), when an external magnetic field is applied along the z-axis at different intensities up to reach the magnetic saturation (Ms) at the core.

MENPs assume a dipole configuration when subjected to an external magnetic field. In the first analysis, 100 nm Ø CoFe/BaTi core/shell MENPs were stimulated by different DC fields. As already demonstrated and here reported, the electric potential generated increases accordingly with the DC applied, due to the MENPs magnetoelectric behavior (Figure 1a–c). Consequently, also the intensity of the electrical heat source, i.e., the electric field (E), is enhanced (Figure 1d–f), leading to the generation of higher local temperatures (Figure 1g–i). Interestingly, if stimulated by a DC field able to elicit the magnetic saturation at the magnetostrictive core (Ms), the temperature on the MENP surface reaches a maximum value of 56 °C (Figure 1i).

**Figure 2:**
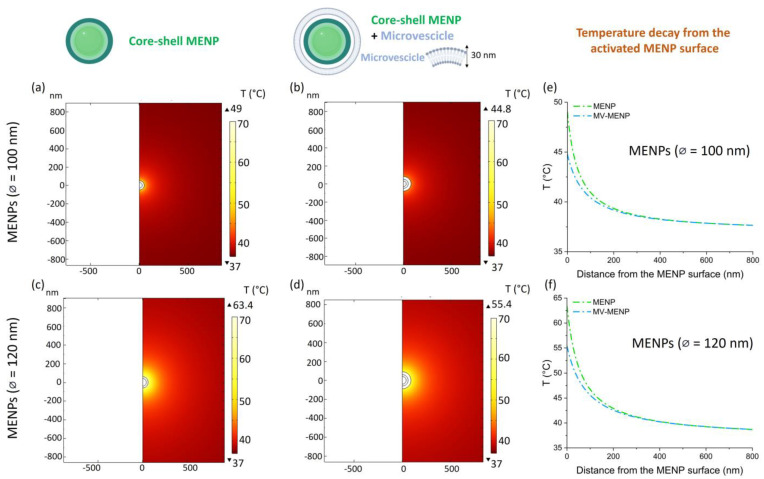
Temperature distribution in the MENP surrounding environment. Color maps showing the temperature distribution in the surrounding extracellular matrix in an axisymmetric 2D model when MENPs with two different diameters (100 nm (**a**,**b**) and 120 nm (**c**,**d**)), without and with the MV-coating, are activated by an external magnetic field at saturation. Graphs (**e**,**f**) show the profiles of temperature decays for all the cases considered.

The localized increase in the temperature on the MENPs surrounding tissue, modeled as the extracellular fluid, when stimulated by an external magnetic field at saturation, has been reported in Figure 2 by varying (i) the core MENPs diameter (while keeping fixed the shell size), (ii) with and without an MV coating of 30 nm of thickness. The reached temperature range is depicted in the color maps of Figure 2a–d, along with the plotted decreasing trend of temperature as a function of the distance from the MENP surface (Figure 2e,f). When 100 nm Ø MENPs were modeled, the maximum temperature reached on the simulated surrounding tissue/fluid was around 50 °C, and an evident effect was detectable, in terms of thermal decay, when MENPs were functionalized with an MV coating, probably due to a damping action of the MV layer that dissipates local heat (Figure 2e). When 120 nm Ø MENPs were computed, the maximum temperature reached in the simulated extracellular fluid was about 65 °C, and the damping effect due to the MV layer was present as well (Figure 2f).

**Figure 3:**
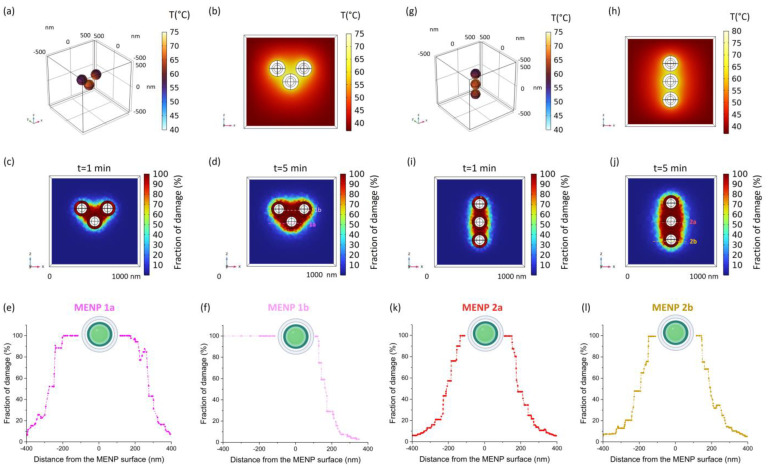
Temperature distribution generated by 3D cluster models. MV-MENP temperature (**a**,**g**), tissue temperature distribution (**b**,**h**), and the fraction of tissue damage at different time points (**c**,**d**,**i**,**j**) generated by 3 MV-MENPs (MENP diameter: 120 nm, MV-thickness: 30 nm) within two different cluster models when they are activated by an external magnetic field at saturation (Ms). Graphs (**e**,**f**,**k**,**l**) show the decay of the fraction of tissue damage with the distance from the MENPs surface.

To model a more realistic experimental scenario, small clusters composed of three 120 nm MV-coated MENPs were modeled in a 3D setting when stimulated with a DC field (=Ms). Two different geometrical configurations were considered. As presented in Figure 3a,g the temperature reached on the external surfaces of the MENPs is subjected to variations depending on their mutual positions. Accordingly, the temperature increase in the extracellular fluid distributes differently based on the cluster configuration (Figure 3b,h). A predictive Arrhenius model of tissue damage was carried out for both cases, and the fraction of damaged tissue is shown in the respective color maps after 1 (Figure 3c,i) and 5 min (Figure 3d,j) of DC magnetic stimulation. As expected, the geometrical distribution of the MENPs-surrounding microenvironment completely damaged (fraction of damage = 100%) by the thermal treatment is different between the two configurations, although, by means of a numerical analysis of each modeled section, the percentages relative to the affected areas were found comparable (12.63% vs. 12.37%).

Since the objective of the current work is to exploit the local temperature increase to denature cytokines linked on the MENPs surface, the fraction of damage in the proximity of the MENPs external layer was derived and plotted (Figure 3e,f,k,l). For each configuration, MENPs in two different positions were considered in order to widen the possible scenario and dampen the effect of cytokine positioning. Interestingly, after 5 min of magnetic stimulation, the fraction of completely damaged microenvironment is well above 80% within 50 nm from all the MENPs surfaces. It is worth noting that this distance encompasses the entire theoretical length of an antibody-cytokine complex bound to the MENP surface.

**Figure 4:**
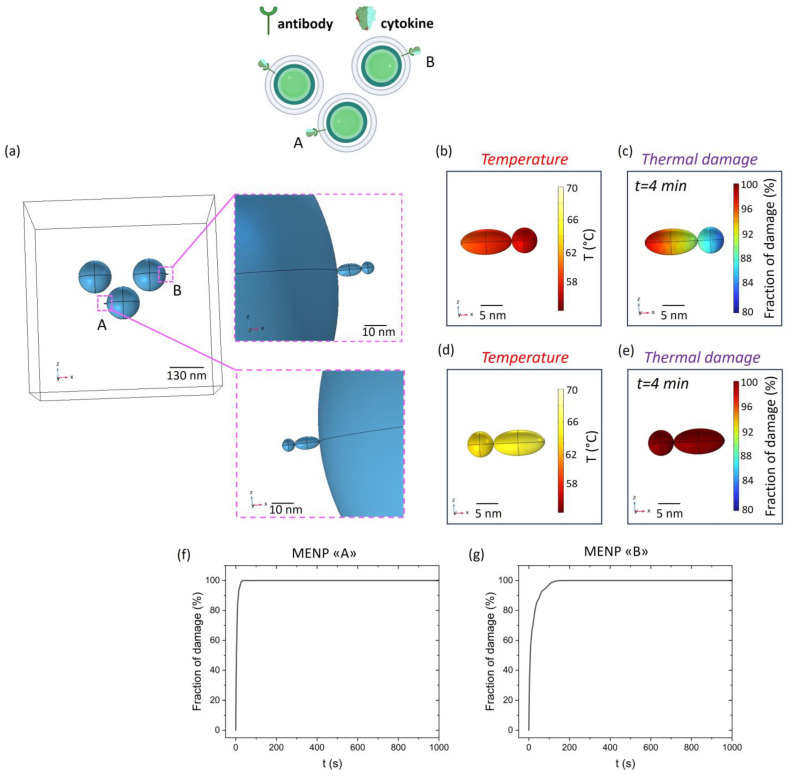
Cytokine-conjugated 3D cluster model. Schematic representation of the two different antibody linking sites considered on the external surface of two distinct MENPs (**a**). Temperature distribution (**b**,**d**) and fraction of damage (**c**,**e**) of a target cytokine captured through an antibody conjugated over the surface of two different MENPs (A, B). MV-coated MENPs are activated with an external magnetic field at saturation. The kinetics of the fraction of damage as a function of the time of MENPs heating are plotted (**f**,**g**).

Based on these results, a further cluster model was implemented in a 3D setting by considering cytokines captured on the MV-coating through a specific antibody. As depicted in Figure 3, 100% thermal damage affects the entire area ideally encompassed by the 3 MENPs; therefore, it is intuitively evident that any captured cytokine within the same area will be subjected to maximum damage. Therefore, to evaluate the effect of the positioning on the efficiency of cytokine denaturation under the stimulus of a DC (=Ms) magnetic field, two different antibody linking sites (Figure 4a) were considered on the external surface of two different MENPs, where the thermal damage rapidly diminishes (Figure 3e,f). As shown in Figure 4b,d, the temperature increase at the cytokine level is slightly different among the two considered positions (A and B). In fact, the damage kinetic is subjected to weak variations: in the more favorable case (Figure 4e,f and Position A), the cytokine is completely damaged within two minutes of simulation, while in the other configuration (Figure 4c,g and Position B), five minutes are needed to reach the same thermal destructive efficacy.

## 3. Discussion

Biological neutralization represents an effective strategy to deplete the organism of endogenous or exogenous harmful molecules. In several immune-mediated diseases, such as rheumatoid arthritis [19], macrophage activation syndrome [20,21,22], or even sepsis-like syndromes [23], it is of primary importance to design therapies able to catch early inflammatory secreted molecules before permanent tissue damage may occur and before the dangerous onset of auto-inducible hypercytokinemia. In the present study, a novel technological approach is presented by exploiting the unique coupled magnetoelectrical features of MENPs to denature cytokines in an in silico framework. In particular, when stimulated by an external magnetic stimulus, MENPs generate very high electric fields (up to 10^5^ V/m) in the surrounding milieu, which decay very quickly with distance. Consequently, the local power density may generate an extremely confined microenvironment heating due to the Joule effect. The MENPs thermal capability of denaturing harmful molecules in the range of tens of nanometres without affecting the surrounding tissue at the macroscale has been here investigated for the first time.

In the current modeling, MENPs are embedded within a biological tissue, which is modeled as an extracellular fluid, the parameters of which are reported in Table 1. MENPs deployment in living tissues could be performed through the vascular network and by an appropriate magnetic confinement, as already demonstrated in a different setting [24]. In spite of the fact that endothelial cells of the capillaries provide a strictly regulated interface between vessels and tissues, translocation of MENPs through the blood-brain barrier has already been achieved by magnetostriction [25]. However, given their size, the modeled MENPs are not suitable to pass the tight junction compartment of the endothelium, but transcytosis can be eased by the applied magnetic fields. Thus, since the scavenging action of MENPs cannot be exerted within the intracellular compartment, MENP localization was simulated in the extracellular compartment, mostly composed of proteins and biological fluids. In this respect, the parameters of such a compartment (i.e., density, dielectric properties, thermal features, and parameters related to the Arrhenius model) were taken into account in the simulation (Table 1). The temperature increase depends on the applied magnetic field intensity. As presented in Figure 1g, no heating is generated with a DC stimulation of 50 mT. Hence, permanent magnets generating such an intensity (or a lower one) could potentially allow to spatial localize MENPs in a specific tissue site and subsequently, upon a proper increase in the DC strength, also allow the exploitation of the heating effect.

The reached temperature depends on the core size, as depicted in Figure 2. This is consistent with our previous findings [15], which report that the ME effect, and therefore the obtained electric field, is maximized by increasing the magnetostrictive phase content. In fact, larger cores transfer a higher strain on the outer piezoelectric shell, which transduces the mechanical signal into a change in charge distribution. A 30 nm-microvesicle coating was then modeled on the surface of the piezoelectric shell to approach a reliable experimental scenario. Coatings can act as effective linkers for antibody attachment [26] and cell-to-cell communication. In addition, biomimetic camouflage of drug-carrier particles with natural cell membranes enhances the stealth properties of the exogenous vectors and extends their permanence in the target tissues [16,27]. The membrane cloaking approach, in fact, preserves the intact proteolipid composition and the set of proteins essential for biointerfacing, endowing the NPs with the desirable functionality of the parental cells. This strategy is particularly useful for drug delivery purposes and relies on the interactions among proteins of the membranes used for particle coating and the surrounding cells in the treated tissue. This approach was proven feasible in an in vivo model of osteoarthritis: coupling glucocorticoid-loaded NPs with a wrapping with CD90+MSCs-derived membranes reactivated chondrocyte proliferation, as already demonstrated for CD90+ MSC alone, and—at the same time—allowed compensation for the poor anti-inflammatory and analgesic properties of the cells by on-site drug release [28,29]. Additionally, NPs camouflaged by macrophage membrane systems have been devised to take advantage of the innate chemotactic ability of macrophages to drive the vector in chronically inflammated sites, a property particularly useful for tumor targeting approaches [30,31] as well as the presence of membrane-bound specific receptors in wound healing approaches [32]. It has to be noted, though, that a camouflage membrane may also represent a barrier to drug delivery, but, on the other hand, tumor-derived microvesicles, used to coat NPs, maintain their ability to activate dendritic cells and thus can foster the development of anti-tumor immunity and, at the same time, improve chemotherapy efficacy by mitigating the possible systemic toxicity of their drug cargo [33,34,35]. In addition, the main caveat of the system resides in the membrane- or microvesicles-derived-coating complexity. Possibly, then, a simplification of the components of cloaking membranes [36] as well as the use of a simpler anti-fouling coating may ease the linking of specific receptors/ligands onto the NPs surfaces to provide and/or enhance unique binding properties [37].

Therefore, for the intended approach, the particle’s affinity toward a defined target molecule should be maximized by an extensive coating with a specific antibody. This could be achieved by providing a coating layer, such as that obtained using bi-functional polyethylene glycol (PEG), in which silane [38] and phosphonate moieties [39] can be added to ensure PEG grafting to the MENP, leaving the other extremity of the molecule available for more specific functionalizations. Alternatively, an MV-derived lipid layer can be envisaged for the same purpose, providing a suitable chemistry that can be applied to link specific antibodies onto the MENP surface [29].

The resulting layers may interfere with the physicochemical properties of the particle [40]. In our simulation, the thickness was set on the basis of previously published experimental works [29]. The temperature reached on the surrounding tissue is lower in the presence of the MV layer because of its dissipative capability.

In the second part of the work, MENP clustering and mutual positioning variation are considered to take into account the intrinsic experimental variability. In our setting, two different clusters are modeled in order to dampen the effect of the cytokine random positioning. Ideally, though, MV-MENPs will be fully decorated to provide target binding sites covering the entire surface, according to [41]. Depending on the possible chemistry to be used for antibody ligation and distribution onto the MV surface, the number of sites per MENP—i.e.; its capture efficiency—could be in the order of several thousand. Of course, this value can be influenced by the MENP shape and size; therefore, future in silico studies will be performed to optimize the cytokine capture efficiency by tuning the particle geometrical features.

As shown in Figure 3, there is a global temperature increase due to interaction phenomena among the MENPs, which should be considered in designing an experimental set-up. Interestingly, although the tissue damage distribution is different among the two configurations, the percentage of tissue undergoing full damage was comparable. It is worth noticing that we demonstrated that a temperature able to cause protein denaturation (above 55 °C) was reached and maintained in a range of 50 nanometers from the MV-MENP outer surface [42,43,44].

The Arrhenius model is generally employed to describe the thermal damage processes of cells and tissues [45]. An Arrhenius plot illustrates the relationship between the logarithm of a reaction rate constant (k) as a function of the inverse of temperature. The activation energy E and the frequency factor A can be then derived. Therefore, a threshold of thermal damage in various tissues can be empirically obtained [18].

It is generally recognized that the Arrhenius model is based on the assumption that protein denaturation is the major cause of thermal damage to tissues and cells. Such a modeling approach is therefore specifically appropriate to our experimental conditions: indeed, the dimensions of the simulated antibody and target cytokine were derived from the literature [46], choosing feasible values for both structures.

More than 90% of the target cytokine will be denatured within 5 min of magnetic stimulation, as shown in Figure 4. The thermic effect that originates from the MENP surface may act at first on the antibody directly linked to the MV-coating and then to the target cytokine. Nonetheless, providing a sufficient amount of time to allow proper antibody-cytokine recognition, the linked target molecule will be kept within the action range of the denaturing temperature, and it will be subjected to the thermic effect as well, even if its capturing antibody will be damaged by the temperature increase.

From the experimental point of view, AC magnetic fields could be adopted to grant a homogenous magnetic field able to keep spatially fixed the MENPs and trigger the temperature increase once they have been pulled to a target tissue, providing larger chances of applicability to in vivo models and to translational medicine. Interestingly, with low frequency and relatively low amplitude AC magnetic fields, the temperature local increase may in principle be tuned in a safer and more controllable manner than other kinds of nanoparticles, which are activated by high power and high-frequency stimulation [47,48]. In this regard, in vivo studies suggested that renal clearance takes care of toxicity elicited by residual MENPs, as already demonstrated/verified for other types of nanoparticles [49]. Furthermore, several studies reported the absence of an inflammatory response in mice [50,51,52]. Indubitably, their clearance kinetics will depend on the surface charge, specific surface groups, and chain-end functionalities [49,53]. Interestingly, neutralization of the target molecule takes place immediately upon application of the proper magnetic field, avoiding the persistency of residual long-lasting cytokine-bonded nano-vectors and their eventual dissociation in other body districts. Therefore, the disabled inflammatory cytokine could also follow the MENPs clearance pathway, which is similar to that of other types of nanoparticles.

Several major issues remain open and need to be tackled in the future; for example, the reverse procedure by which MENPs will be driven back to the vascular tree from their localized site of action in order to take advantage of the above-mentioned clearance kinetics has to be defined. At the same time, punctual experimental observations need to be undertaken to evaluate the fate of the denatured target cytokine(s), both at the degradation site and in the surrounding tissues, given that thermal will affect the biological, chemical, and physical properties of the proteins themselves. Lastly, the effectiveness of the tailored MENPs will have to be coupled with the duration and intensity of the hyper-inflammatory stimulus under treatment.

## 4. Materials and Methods

### 4.1. MENPs Multiphysics Model

COMSOL Multiphysics 5.6 “www.comsol.com (accessed on 6 December 2024)” was adopted to model MENPs with different configurations, as described below. In all cases, the following subdomains are present: (i) the magnetostrictive MENPs core; (ii) the piezoelectric MENPs shell; and (iii) the surrounding extracellular fluid. Tables resuming the material properties of the CoFe core and the BaTi shell were already reported in our previous studies [15,16] and in Tables S1 and S2. The properties related to the extracellular fluid are reported in Table 1.

#### 4.1.1. Two-Dimensional MENPs Model

MENPs were modeled by an axisymmetric bi-dimensional (2D) model of core-shell spheres and subjected to different static (DC) external magnetic fields (50 mT, 300 mT, 1 T (Ms)) directed along the z-axis (Figure 1). The last applied DC magnetic field is strong enough to reach magnetic saturation in the core (Ms) for the MENPs considered in the present study, whose magnetic features are reported in Table S1. The diameter of the core of the MENPs varied (60 nm or 80 nm) (Figure 2), while, for all configurations, the shell thickness was set at 20 nm, previously acknowledged as the optimal one [15]. MENPs were functionalized with a 30 nm thick MV-coating, whose properties are described in Table 1. For all tested conditions, temperature increases were derived, and the corresponding kinetics were plotted through the Origin Pro. 8.5 software (Origin Lab Co., Northampton, MA, USA).

#### 4.1.2. Three-Dimensional MENPs Cluster Model

Two different cluster configurations were designed by adopting a 3D model (Figure 3). Each cluster is composed of three different MV-coated MENPs (MENP diameter = 120 nm) positioned, in both configurations, at a distance of about 30 nm. MENPs were stimulated with a high-strength (=Ms) DC bias magnetic field directed along the z-axis. The temperature increase and thermal damage were derived. The thermal damage kinetic was plotted as above mentioned.

#### 4.1.3. Cytokine-Conjugated 3D Cluster Model

A cluster of 3 MV-coated MENPs conjugated with a target cytokine was designed. In particular, an antibody was modeled as an ellipsoid (major axis: 10 nm; minor axis: 5 nm) and the attached target cytokine as a sphere (diameter: 5 nm). Dimensions of the depicted conjugated complex were averaged on the basis of previously published measurements of its components [46]. The complex antibody-cytokine was located and modeled in two different positions to evaluate the kinetics of thermal damage in possible different configurations. The temperature increase and thermal damage at the level of the antibody-cytokine complex were derived and plotted as previously indicated.

### 4.2. COMSOL Multiphysics Equations

In the following sections, the equations governing the (*i*) MENPs magnetoelectric behavior, (*ii*) temperature increase, and (*iii*) tissue damage generated by MENPs activations are solved by using COMSOL Multiphysics [15].

#### 4.2.1. Magnetoelectric Nanoparticles Model

The magnetoelectric behavior is modeled through stationary studies by using the Magnetic Fields, Solid Mechanics, and Electrostatics COMSOL physics and by means of the coupled Magnetostriction and Piezoelectric Multiphysics. Details about the mathematical equations governing the model are derived from published literature [15], which are reported in detail.

#### 4.2.2. Localized Temperature Distribution

In the present study, the Electric Currents physics of the AC/DC module and the Heat Transfer in Biological Tissue physics of the Heat Transfer module of COMSOL Multiphysics have been coupled through the Electromagnetic Heat Source Multiphysics to implement transient studies in 2D and 3D configurations. Through this analysis, the localized temperature increase generated when MENPs are activated has been evaluated.

Firstly, the electric dipole obtained from the stationary studies described above was set on the surface of the BaTi shell through the Electric Current physic. The electric field intensity (*E*) and the current density (*J*) within the surrounding tissue can be derived from Equations (1) and (2):

(1) E=−∇V

(2) J=σE

The power density that induces local tissue heating is defined as the product of current density (*J*) and electric field intensity (*E*), according to Equation (3):

(3) $$JE=\sigma E^{2}$$

The Heat Transfer in the Biological Tissue module is adopted to study the MENPs effect in a confined biological environment in terms of localized heating. The time-dependent power density conversion equation is described below according to Equation (4):

(4) $$\rho C_{p}\frac{\delta T}{\delta t}-\nabla \left(k\nabla T\right)=Qext$$

Specifically, ρ is the tissue density (kg/m^3^); $C_{p}$ is the tissue specific heat (J/(kg·K)), *k* is its thermal conductivity (W/(m·K)), while Qext is the heat source (W/m^3^). In particular, Qext represents the Joule heating induced by the high electric field generated by the MENPs piezoelectric shell, defined in Equation (3). The initial temperature is set at 37 °C in all domains. The simulations were carried out considering a time range of 10 min.

**Table 1:** Electrical and thermal properties of the biological domains considered.

Domain	Electrical Conductivity (S/m)	Relative Permittivity	Density (kg/m^3^)	Thermal Conductivity (W/m·K)	Tissue-Specific Heat Capacity (J/(kg·K))	Activation Energy (kJ/mol)	Frequency Factor (1/s)
Extracellular Fluid	2 [54]	109 [54]	1006 [54]	0.60 [54]	3997 [16]	281 [45]	2.97 × 10^42^ [45]
MV-Coating	0.3 [55]	80 [56]	1380 [57]	0.547 [56]	3890 [56]	281 [45]	2.97 × 10^42^ [45]
Antibody- Cytokine Complex	0.16 [58]	3.23 [59]	1350 [60]	0.3 [61]	1200 [62]	178 [45,63]	1.42 × 10^26^ [45]

#### 4.2.3. Arrhenius Model

The induced thermal damage has been computed using the first-order Arrhenius equation through the Bioheat Transfer Physic, which associates damage with temperature exposure time. The Arrhenius model is a simple first-order irreversible reaction kinetic model widely used to predict both protein denaturation and cell injury. It calculates the fraction of tissue damage (*α*) during the thermal process based on Equation (5):

(5) $$\frac{d\alpha }{dt}=A\exp\left(-\frac{∆E}{RT}\right)$$

where *R* is the universal gas constant, *A* is the frequency factor (1/s), and *E* is the activation energy (kJ/mol) for the irreversible damage reaction. The two parameters (*E* and *A*) used to describe the kinetics of thermally induced protein denaturation are related to the reaction activation enthalpy (*E*) and to the reaction entropy (*A*), according to the Eyring–Polyani equation [63]. The parameters adopted to model the thermal damage in the different biological structures encountered (MV-based coating, the antibody, the target cytokine, and the surrounding extracellular matrix) are resumed in Table 1. The simulations lasted for up to 10 min.

## 5. Conclusions

The herewith presented data exploits, for the first time, the properties of novel magnetoelectric nanoparticles acting as new cytokine inhibitors; MENPs were modeled and envisaged as sc [avengers of specific harmful molecules in the context of “cytokine-cascade”-affected tissues, with the additional advantage of bypassing the cross-toxicity effects persisting in the actual therapies, in which cytokine inhibitors are systemically administered and are unable to be crammed in a confined tissue localization.

MENPs electromagnetic and physical features as well as a proper biomimicry coating were here tailored on the basis of biological parameters and of the needs of their prospective applications in vitro and in vivo. Indeed, in advance of any possible therapeutic application, the most suitable antibody-decorative chemistry as well as the scavenging efficiency of target molecules will have to be thoroughly assessed on the bench.

In this perspective, by predicting the best array of parameters and conditions, our study provides new insights for the establishment of experimental procedures and conditions to optimize the initial trial-and-error research pathway and its relative costs, necessary to transfer this novel MENPs feature into a clinical application.

## Data Availability

The original contributions presented in this study are included in the article. Further inquiries can be directed to the corresponding author.

## Supplementary Materials

The following supporting information can be downloaded at: https://www.mdpi.com/article/10.3390/ijms252413591/s1. References [15,64,65,66,67,68] are cited in the Supplementary Material.

## References

[1] Hanada T., Yoshimura A. (2002). Regulation of Cytokine Signaling and Inflammation. Cytokine Growth Factor Rev..

[2] Lima A.C., Cunha C., Carvalho A., Ferreira H., Neves N.M. (2018). Interleukin-6 Neutralization by Antibodies Immobilized at the Surface of Polymeric Nanoparticles as a Therapeutic Strategy for Arthritic Diseases. ACS Appl. Mater. Interfaces.

[3] Ritz M.A., Jost R. (2001). Severe Pneumococcal Pneumonia Following Treatment with Infliximab for Crohn’s Disease. Inflamm. Bowel Dis..

[4] Smith D., Letendre S. (2002). Viral Pneumonia as a Serious Complication of Etanercept Therapy. Ann. Intern. Med..

[5] Marotte H., Charrin J.E., Miossec P. (2001). Infliximab-Induced Aseptic Meningitis. Lancet.

[6] Baghai M., Osmon D.R., Wolk D.M., Wold L.E., Haidukewych G.J., Matteson E.L. (2001). Fatal Sepsis in a Patient with Rheumatoid Arthritis Treated with Etanercept. Mayo Clin. Proc..

[7] Riminton S., Pearce N., Antony B. (2002). Tuberculosis and Treatment with Infliximab. N. Engl. J. Med..

[8] Warris A., Bjorneklett A., Gaustad P. (2001). Invasive Pulmonary Aspergillosis Associated with Infliximab Therapy. N. Engl. J. Med..

[9] Thamphiwatana S., Angsantikul P., Escajadillo T., Zhang Q., Olson J., Luk B.T., Zhang S., Fang R.H., Gao W., Nizet V. (2017). Macrophage-like Nanoparticles Concurrently Absorbing Endotoxins and Proinflammatory Cytokines for Sepsis Management. Proc. Natl. Acad. Sci. USA.

[10] Rao L., Xia S., Xu W., Tian R., Yu G., Gu C., Pan P., Meng Q.-F., Cai X., Qu D. (2020). Decoy Nanoparticles Protect against COVID-19 by Concurrently Adsorbing Viruses and Inflammatory Cytokines. Proc. Natl. Acad. Sci. USA.

[11] Hu C.-M.J., Fang R.H., Copp J., Luk B.T., Zhang L. (2013). A Biomimetic Nanosponge that Absorbs Pore-Forming Toxins. Nat. Nanotechnol..

[12] Hasan M., Choi J., Akter H., Kang H., Ahn M., Lee S. (2024). Antibody-Conjugated Magnetic Nanoparticle Therapy for Inhibiting T-Cell Mediated Inflammation. Adv. Sci..

[13] Wang P., Zhang E., Toledo D., Smith I.T., Navarrete B., Furman N., Hernandez A.F., Telusma M., McDaniel D., Liang P. (2020). Colossal Magnetoelectric Effect in Core–Shell Magnetoelectric Nanoparticles. Nano Lett..

[14] Marrella A., Suarato G., Fiocchi S., Chiaramello E., Bonato M., Parazzini M., Ravazzani P. (2023). Magnetoelectric Nanoparticles Shape Modulates Their Electrical Output. Front. Bioeng. Biotechnol..

[15] Fiocchi S., Chiaramello E., Marrella A., Suarato G., Bonato M., Parazzini M., Ravazzani P. (2022). Modeling of Core-Shell Magneto-Electric Nanoparticles for Biomedical Applications: Effect of Composition, Dimension, and Magnetic Field Features on Magnetoelectric Response. PLoS ONE.

[16] Fernández-Borbolla A., García-Hevia L., Fanarraga M.L. (2024). Cell Membrane-Coated Nanoparticles for Precision Medicine: A Comprehensive Review of Coating Techniques for Tissue-Specific Therapeutics. Int. J. Mol. Sci..

[17] Ben-Akiva E., Meyer R.A., Yu H., Smith J.T., Pardoll D.M., Green J.J. (2020). Biomimetic Anisotropic Polymeric Nanoparticles Coated with Red Blood Cell Membranes for Enhanced Circulation and Toxin Removal. Sci. Adv..

[18] Dewey W.C. (1994). Arrhenius Relationships from the Molecule and Cell to the Clinic. Int. J. Hyperth..

[19] McInnes I.B., Schett G. (2011). The Pathogenesis of Rheumatoid Arthritis. N. Engl. J. Med..

[20] Bracaglia C., Prencipe G., De Benedetti F. (2017). Macrophage Activation Syndrome: Different Mechanisms Leading to a One Clinical Syndrome. Pediatr. Rheumatol..

[21] Deane S., Selmi C., Teuber S.S., Gershwin M.E. (2010). Macrophage Activation Syndrome in Autoimmune Disease. Int. Arch. Allergy Immunol..

[22] Lerkvaleekul B., Vilaiyuk S. (2018). Macrophage Activation Syndrome: Early Diagnosis Is Key. Open Access Rheumatol. Res. Rev..

[23] Siddall E., Khatri M., Radhakrishnan J. (2017). Capillary Leak Syndrome: Etiologies, Pathophysiology, and Management. Kidney Int..

[24] Chen S., Chen E., Guan X., Li J., Qin A., Wang C., Fu X., Huang C., Li J., Tang Y. (2024). Magnetically Controlled Nanorobots Induced Oriented and Rapid Clearance of the Cytokine Storm for Acute Lung Injury Therapy. Colloids Surf. B Biointerfaces.

[25] Mhambi S., Fisher D., Tchokonte M.B.T., Dube A. (2021). Permeation Challenges of Drugs for Treatment of Neurological Tuberculosis and HIV and the Application of Magneto-Electric Nanoparticle Drug Delivery Systems. Pharmaceutics.

[26] Tang Q., Sun S., Wang P., Sun L., Wang Y., Zhang L., Xu M., Chen J., Wu R., Zhang J. (2023). Genetically Engineering Cell Membrane-Coated BTO Nanoparticles for MMP2-Activated Piezocatalysis-Immunotherapy. Adv. Mater..

[27] Wu M., Le W., Mei T., Wang Y., Chen B., Liu Z., Xue C. (2019). Cell Membrane Camouflaged Nanoparticles: A New Biomimetic Platform for Cancer Photothermal Therapy. Int. J. Nanomed..

[28] Li Y., Tu Q., Xie D., Chen S., Gao K., Xu X., Zhang Z., Mei X. (2022). Triamcinolone Acetonide-Loaded Nanoparticles Encapsulated by CD90^+^ MCSs-Derived Microvesicles Drive Anti-Inflammatory Properties and Promote Cartilage Regeneration after Osteoarthritis. J. Nanobiotechnol..

[29] Li R., He Y., Zhu Y., Jiang L., Zhang S., Qin J., Wu Q., Dai W., Shen S., Pang Z. (2018). Route to Rheumatoid Arthritis by Macrophage-Derived Microvesicle-Coated Nanoparticles. Nano Lett..

[30] Zhang Y., Cai K., Li C., Guo Q., Chen Q., He X., Liu L., Zhang Y., Lu Y., Chen X. (2018). Macrophage-Membrane-Coated Nanoparticles for Tumor-Targeted Chemotherapy. Nano Lett..

[31] Wang Y., Zhang D., Jia M., Zheng X., Liu Y., Wang C., Lei F., Niu H., Li C. (2022). ZIF-8 Nanoparticles Coated with Macrophage-Derived Microvesicles for Sustained, Targeted Delivery of Dexamethasone to Arthritic Joints. J. Drug Target..

[32] Peng Z., Zhang X., Yuan L., Li T., Chen Y., Tian H., Ma D., Deng J., Qi X., Yin X. (2021). Integrated Endotoxin-Adsorption and Antibacterial Properties of Platelet-Membrane-Coated Copper Silicate Hollow Microspheres for Wound Healing. J. Nanobiotechnol..

[33] Zhang S., Zheng B., Wei Y., Liu Y., Yang L., Qiu Y., Su J., Qiu M. (2024). Bioinspired Ginsenoside Rg3 PLGA Nanoparticles Coated with Tumor-Derived Microvesicles to Improve Chemotherapy Efficacy and Alleviate Toxicity. Biomater. Sci..

[34] Zhou Z., Zhang S., Xue N. (2023). Research Progress of Cancer Cell Membrane Coated Nanoparticles for the Diagnosis and Therapy of Breast Cancer. Front. Oncol..

[35] Imran M., Jha L.A., Hasan N., Shrestha J., Pangeni R., Parvez N., Mohammed Y., Jha S.K., Paudel K.R. (2022). “Nanodecoys”—Future of Drug Delivery by Encapsulating Nanoparticles in Natural Cell Membranes. Int. J. Pharm..

[36] Rampado R., Caliceti P., Agostini M. (2022). Latest Advances in Biomimetic Cell Membrane-Coated and Membrane-Derived Nanovectors for Biomedical Applications. Nanomaterials.

[37] Zhang H., Chiao M. (2015). Anti-Fouling Coatings of Poly(Dimethylsiloxane) Devices for Biological and Biomedical Applications. J. Med. Biol. Eng..

[38] Dempsey C., Lee I., Cowan K.R., Suh J. (2013). Coating Barium Titanate Nanoparticles with Polyethylenimine Improves Cellular Uptake and Allows for Coupled Imaging and Gene Delivery. Colloids Surf. B Biointerfaces.

[39] Pa̧zik R., Andersson R., Kȩpiński L., Nedelec J.-M., Kessler V.G., Seisenbaeva G.A. (2011). Surface Functionalization of the Metal Oxide Nanoparticles with Biologically Active Molecules Containing Phosphonate Moieties. Case Study of BaTiO_3_. J. Phys. Chem. C.

[40] Sood A., Desseigne M., Dev A., Maurizi L., Kumar A., Millot N., Han S.S. (2023). A Comprehensive Review on Barium Titanate Nanoparticles as a Persuasive Piezoelectric Material for Biomedical Applications: Prospects and Challenges. Small.

[41] Munir A., Zhu Z., Wang J., Zhou H.S. (2014). FEM Analysis of Magnetic Agitation for Tagging Biomolecules with Magnetic Nanoparticles in a Microfluidic System. Sens. Actuators B Chem..

[42] Narhi L.O., Philo J.S., Li T., Zhang M., Samal B., Arakawa T. (1996). Induction of α-Helix in the β-Sheet Protein Tumor Necrosis Factor-α: Thermal-and Trifluoroethanol-Induced Denaturation at Neutral pH. Biochemistry.

[43] Gu L.C., Erdös E.A., Chiang H.-S., Calderwood T., Tsai K., Visor G.C., Duffy J., Hsu W.-C., Foster L.C. (1991). Stability of Interleukin 1β (IL-1β) in Aqueous Solution: Analytical Methods, Kinetics, Products, and Solution Formulation Implications. Pharm. Res..

[44] Schön A., Clarkson B.R., Jaime M., Freire E. (2017). Temperature Stability of Proteins: Analysis of Irreversible Denaturation Using Isothermal Calorimetry. Proteins Struct. Funct. Bioinform..

[45] Qin Z., Balasubramanian S.K., Wolkers W.F., Pearce J.A., Bischof J.C. (2014). Correlated Parameter Fit of Arrhenius Model for Thermal Denaturation of Proteins and Cells. Ann. Biomed. Eng..

[46] Erickson H.P. (2009). Size and Shape of Protein Molecules at the Nanometer Level Determined by Sedimentation, Gel Filtration, and Electron Microscopy. Biol. Proced. Online.

[47] You D., Chen T., Liu G. (2023). Multiphysics Modeling of Plasmonic Photothermal Therapy. Therm. Sci. Eng. Prog..

[48] Lio G.E., Palermo G., De Luca A., Caputo R. (2020). Numerical Modeling of Active Thermo-Plasmonics Experiments. arXiv.

[49] Kolishetti N., Alexis F., Pridgen E.M., Farokhzad O.C. (2011). Biodistribution and Pharmacokinetics of Nanoprobes. Nanoplatfor-Based Mol. Imaging.

[50] Kaushik A., Jayant R.D., Nikkhah-Moshaie R., Bhardwaj V., Roy U., Huang Z., Ruiz A., Yndart A., Atluri V., El-Hage N. (2016). Magnetically Guided Central Nervous System Delivery and Toxicity Evaluation of Magneto-Electric Nanocarriers. Sci. Rep..

[51] Kozielski K.L., Jahanshahi A., Gilbert H.B., Yu Y., Erin Ö., Francisco D., Alosaimi F., Temel Y., Sitti M. (2021). Nonresonant Powering of Injectable Nanoelectrodes Enables Wireless Deep Brain Stimulation in Freely Moving Mice. Sci. Adv..

[52] Nguyen T., Gao J., Wang P., Nagesetti A., Andrews P., Masood S., Vriesman Z., Liang P., Khizroev S., Jin X. (2021). In Vivo Wireless Brain Stimulation via Non-Invasive and Targeted Delivery of Magnetoelectric Nanoparticles. Neurotherapeutics.

[53] Kolishetti N., Vashist A., Arias A.Y., Atluri V., Dhar S., Nair M. (2022). Recent Advances, Status, and Opportunities of Magneto-Electric Nanocarriers for Biomedical Applications. Mol. Asp. Med..

[54] (2018). IT’S Foundation Tissue Properties Database 4.0.

[55] Towhidi L., Khodadadi D., Maimari N., Pedrigi R.M., Ip H., Kis Z., Kwak B.R., Petrova T.W., Delorenzi M., Krams R. (2016). Comparison between Direct and Reverse Electroporation of Cells in Situ: A Simulation Study. Physiol. Rep..

[56] Yan Z., Hao C., Yin L., Liu K., Qiu J. (2021). Simulation of the Influence of Temperature on the Dynamic Process of Electroporation Based on Finite Element Analysis. IEEE Trans. Plasma Sci..

[57] Yurinskaya V.E., Vereninov I.A., Vereninov A.A. (2019). A Tool for Computation of Changes in Na^+^, K^+^, Cl^−^ Channels and Transporters Due to Apoptosis by Data on Cell Ion and Water Content Alteration. Front. Cell Dev. Biol..

[58] Ayadi M., Leuliet J., Chopard F., Berthou M., Lebouche M. (2004). Electrical Conductivity of Whey Protein Deposit: Xanthan Gum Effect on Temperature Dependency. Food Bioprod. Process..

[59] Amin M., Küpper J. (2020). Variations in Proteins Dielectric Constants. ChemistryOpen.

[60] Fischer H., Polikarpov I., Craievich A.F. (2004). Average Protein Density Is a Molecular-weight-dependent Function. Protein Sci..

[61] Yamato T., Wang T., Sugiura W., Laprévote O., Katagiri T. (2022). Computational Study on the Thermal Conductivity of a Protein. J. Phys. Chem. B.

[62] Högg E., Rauh C. (2023). Towards a Better Understanding of Texturization during High-Moisture Extrusion (HME)—Part I: Modeling the Texturability of Plant-Based Proteins. Foods.

[63] Huang L., Hwang A., Phillips J. (2011). Effect of Temperature on Microbial Growth Rate–Mathematical Analysis: The Arrhenius and Eyring–Polanyi Connections. J. Food Sci..

[64] Chinnasamy C.N., Senoue M., Jeyadevan B., Perales-Perez O., Shinoda K., Tohji K. (2003). Synthesis of size-controlled cobalt ferrite particles with high coercivity and squareness ratio. J. Colloid Interface Sci..

[65] Betal S., Shrestha B., Dutta M., Cotica L.F., Khachatryan E., Nash K., Tang L., Bhalla A.S., Guo R. (2016). Magneto-elasto-electroporation (MEEP): In-Vitro visualization and numerical characteristics. Sci Rep.

[66] Zhao X., Feng M., Liu M., Hua J., Ma J., Wu L., Xu H., Wang A.P., Li H.B. (2018). Electric-field tuning of magnetic anisotropy in the artificial multiferroic Fe3O4/PMN–PT heterostructure. Mater. Res. Lett..

[67] Kurian M., Thankachan S., Nair D.S., EK A., Babu A., Thomas A., Krishna KT B. (2015). Structural, magnetic, and acidic properties of cobalt ferrite nanoparticles synthesised by wet chemical methods. J. Adv. Ceram..

[68] Ananth Subray P.V., Hanumagowda B.N., Raju CS K., Varma SV K., Jagdish P., Yook S.J., Shah N.A. (2023). Analytical analysis of inclined three-layered composite channel with cobalt ferrite nanoparticles and Hall current in Darcy medium. Propuls. Power Res..

