# Supplementary material for: Temperature-Dependent Cytokine Neutralization Induced by Magnetoelectric Nanoparticles: An In Silico Study
Source: Int J Mol Sci. 2024 Dec 19;25(24):13591. doi: 10.3390/ijms252413591 (PMC11678122; doi:10.3390/ijms252413591)
Supplement: Supplementary file 1 [file ijms-25-13591-s001.zip › ijms-3359224-supplementary.pdf]

*Supporting Information*

# Temperature-Dependent Cytokine Neutralization Induced by Magnetoelectric Nanoparticles: An In Silico Study

**Alessandra Marrella [1,*], Paolo Giannoni [2], Martina Lenzuni [1], Giulia Suarato [1], Serena Fiocchi [1], Emma Chiaramello [1] and Paolo Ravazzani [1]**

[1] Institute of Electronics, Computer and Telecommunication Engineering (IEIIT), National Research Council of Italy (CNR), 20133 Milan, Italy; martina.lenzuni@cnr.it (M.L.); giulia.suarato@cnr.it (G.S.); serena.fiocchi@cnr.it (S.F.); emma.chiaramello@cnr.it (E.C.); paolo.ravazzani@cnr.it (P.R.)

[2] Department of Experimental Medicine, Biology Section, University of Genoa, 16132 Genoa, Italy; paolo.giannoni@unige.it

* Correspondence: alessandra.marrella@cnr.it

**Table S1. MENPs core properties**

| | Symbol | Value | Reference |
|---|---|---|---|
| Magnetic Saturation [A/m] | $M_S$ | 3.69*10$^5$ | Chinnasamy *et al.* [1] |
| Saturation magnetostriction [ppm] | $\lambda_S$ | -200 | Betal *et al.* [2], Zhao *et al.* [3]*19/12/2024 14:59:00* |
| Density [kg/m$^3$] | $\rho$ | 5200 | Kurian *et al.* [4], COMSOL Library |
| Initial magnetic susceptibility | $\chi_0$ | 3 | Betal *et al.* [2], Fiocchi *et al.* [5] |
| Electrical conductivity [S/m] | $\sigma$ | 5.2*10$^6$ | COMSOL Library |
| Poisson ratio | $\nu$ | 0.48 | Zhao *et al.* [3] |
| Young's modulus [GPa] | $E$ | 230 | Zhao *et al.* [3] |
| Relative permeability | $\mu_r$ | 1 | COMSOL Library |
| Thermal conductivity [W/mK] | $K$ | 3.7 | Subray *et al.* [6] |
| Tissue-specific heat capacity [J/(kgK)] | $Cp$ | 700 | Subray *et al.* [6] |

**Table S2. MENPs shell properties (from COMSOL library)**

| | Symbol | Value |
|---|---|---|
| Density [kg/m³] | $\rho$ | 5700 |
| Thermal conductivity [W/mK] | $k$ | 2.61 |
| Tissue-specific heat capacity [J/(kgK)] | $C_p$ | 400 |
| Relative permittivity | $\{\varepsilon r_{11}; \varepsilon r_{22}; \varepsilon r_{33}\}$ | {1115.1; 1115.1; 1251.3} |
| Electrical conductivity [Sm⁻¹] | $\sigma$ | 178.5 |
| Elasticity matrix, Voigt notation [GPa] | *{cE11; cE12; cE22; cE13; cE23; cE33; cE14; cE24; cE34; cE44; cE15; cE25; cE35; cE45; cE55; cE16; cE26; cE36; cE46; cE56; cE66}* | {150.4; 65.6; 150.4;65.; 65.9; 145.5; 0; 0;0;43.9;0; 0; 0; 0;43.9; 0; 0; 0; 0; 0; 42.4} |
| Piezoelectric coupling matrix, Voigt notation [C/m²] | *{eES11; eES21; eES31; eES12; eES22; eES32; eES12; eES23; eES33; eES14; eES24; eES34; eES15; eES25; eES35; eES16; eES26; eES36}* | {0; 0; -4.32; 0; 0; -4.32; 0; 0; 17.4; 0; 11.4; 0; 11.4; 0; 0; 0; 0; 0} |

## References


1. Chinnasamy, C. N. *et al.* Synthesis of size-controlled cobalt ferrite particles with high coercivity and squareness ratio. *J. Colloid Interface Sci.* **263**, 80–83 (2003).
2. Betal, S. *et al.* Magneto-elasto-electroporation (MEEP): in-vitro visualization and numerical characteristics. *Sci Rep* **6**, (2016).
3. Zhao, X. *et al.* Electric-field tuning of magnetic anisotropy in the artificial multiferroic Fe3O4/PMN–PT heterostructure. *Mater. Res. Lett.* **6**, 592–597 (2018).
4. Kurian, M. *et al.* Structural, magnetic, and acidic properties of cobalt ferrite nanoparticles synthesised by wet chemical methods. *J. Adv. Ceram.* **4**, 199–205 (2015).
5. Fiocchi, S. *et al.* Modeling of core-shell magneto-electric nanoparticles for biomedical applications: Effect of composition, dimension, and magnetic field features on magnetoelectric response. *PLOS ONE* **17**, e0274676 (2022).
6. Subray, P. A. *et al.* Analytical analysis of inclined three-layered composite channel with cobalt ferrite nanoparticles and Hall current in Darcy medium. *Propuls. Power Res.* **12**, 523–538 (2023).
